# Acute Rheumatism

**Published:** 1886-10-20

**Authors:** 


**Acute Rheumatism.**—If you wish to impress a new patient favorably by giving relief quickly, give salicylate of soda, ʒ ij daily.

The best results as to preventing heart diseases are obtained by using the alkaline treatment, giving ℥ j carbonate of potash daily till a decided remission occurs; then diminishing the dose one-third each day till it can be omitted entirely.

Iodide of potassium is of particular value when an acute attack has spent its force, but threatens to become chronic. Give ʒ ss to ʒ j daily.

If the disease lingers in a single joint, wrap it in flannel soaked in cod-liver oil.

Remember that stiffness of the joints remaining may be due to disuse; and message will be needed, as it is after a fracture. Knead the muscles well, and bend the joints forcibly in every direction, then rub well with hot camphor liniment, and the joints will soon become supple.—*Medical World.*

